# Desiccation plasticity in the embryonic life histories of non-annual rivulid species

**DOI:** 10.1186/2041-9139-5-16

**Published:** 2014-04-29

**Authors:** Irma Varela-Lasheras, Tom JM Van Dooren

**Affiliations:** 1 Naturalis Biodiversity Center, Darwinweg 2, Leiden 2333 CR, The Netherlands; 2 Current address: Instituto Gulbenkian de Ciência, Rua da Quinta Grande 6, 2780-156 Oeiras, Portugal; 3 CNRS/UPMC/UPEC/UPD/IRD/INRA–UMR 7618 Institute of Ecology and Environmental Sciences Paris (iEES), Université Pierre et Marie Curie, Case 237, 7 Quai St Bernard, 75005 Paris, France

**Keywords:** Killifish, Quiescence, Diapause, Heterokairy, Plasticity evolution, Genetic assimilation, Multi-state modelling, Survival analysis, Lagged responses

## Abstract

**Background:**

Diapause is a developmental arrest present in annual killifish, whose eggs are able to survive long periods of desiccation when the temporary ponds they inhabit dry up. Diapause can occur in three different developmental stages. These differ, within and between species, in their responsiveness to different environmental cues. A role of developmental plasticity and genetic assimilation in diapause evolution has been previously suggested but not experimentally explored. We investigated whether plastic developmental delays or arrests provoked by an unusual and extreme environment could be the ancestral condition for diapause. This would be in agreement with plasticity evolution playing a role in the emergence of diapause in this group. We have used a comparative experimental approach and exposed embryos of non-annual killifish belonging to five different species from the former genus *Rivulus* to brief periods of desiccation. We have estimated effects on developmental and mortality rates during and after the desiccation treatment.

**Results:**

Embryos of these non-annual rivulids decreased their developmental rates in early stages of development in response to desiccation and this effect persisted after the treatment. Two pairs of two different species had sufficient sample sizes to investigate rates of development in later stages well. In one of these, we found cohorts of embryos in the latest stages of development that did not hatch over a period of more than 1 month without mortality. Several properties of this arrest are also used to characterize diapause III in annual killifish. Such a cohort is present in control conditions and increases in frequency in the desiccation treatment.

**Conclusions:**

The presence of plasticity for developmental timing and a prolonged developmental arrest in non-annual rivulids, suggest that a plastic developmental delay or diapause might have been present in the shared ancestor of annual and non-annual South American killifish and that the evolution of plasticity could have played a role in the emergence of the diapauses. Further comparative experimental studies and field research are needed to better understand how diapause and its plasticity evolved in this group.

## Background

Phenotypic plasticity is the ability of organisms to produce different phenotypes in different environments. A key concept regarding plasticity is the genotypic reaction norm, the range of phenotypes which a single genotype can produce [1]. If there is heritable variation for plasticity, selection can act on its phenotypic effects across the environmental range (for example, the slope of the reaction norm). The evolution of plasticity and whether it is maintained or not are thought to be mainly affected by the amount of environmental variability, the predictability of the selection environment, and the costs of plasticity [2]. In a variable environment where no environmental cues convey reliable information about the future selection environment, bet-hedging strategies [3], or genetic polymorphism [4,5] are expected to evolve. When environmental cues can be used to predict the future selection environment, plasticity is expected to be favored and maintained [2]. However, changes in the variance of environmental conditions or in the strength of selection can reduce the relevance of reacting strongly to a cue. Plasticity would then be reduced and potentially lost as a result of its fitness costs [6,7]. In this case, a phenotype initially produced by a plastic response would partially or completely lose its dependency on the environment and become regularly expressed, a process known as genetic assimilation [8]. In a scenario where a variable environment becomes altered [9], some plasticity variation already present allows tracking the environmental change by an overall increase in plasticity. This can then be followed by genetic assimilation, where plasticity or reaction norm slope is reduced again and reaction norm elevation changes relative to the initial environment [8,9]. Plasticity does not need to disappear completely, however, if the altered environment is variable as well.

While the evolutionary significance of plasticity and genetic assimilation have been much discussed, theoretical models [2,9,10] and empirical evidence have accumulated in support of their role in phenotypic diversification and speciation [10-12]. Selection experiments have analyzed the heritability, genetic architecture, and evolutionary dynamics of plasticity [13]. Comparative studies have provided evidence in agreement with plasticity evolution and genetic assimilation occurring in nature (for example, [14]). These studies focused on the reconstruction of ancestral reaction norms, and on the comparison of plasticity between closely related taxa or between ancestral and derived species [14-21]. They have been generally based on the prediction that phenotypic changes by ancestral plasticity in the altered environment should be in the direction of the assimilated phenotype [10,11]. Thus, a species in the process of canalizing a certain phenotype through genetic assimilation, could still exhibit some degree of plasticity in the same direction as the ancestor or as other closely related species.

Heterokairy, plasticity for developmental timing [22], is widespread in multicellular organisms. Embryos respond to cues by accelerating, delaying, or even arresting overall development or by changing relative timings of different processes. Diapause, a complete developmental arrest of variable duration, occurs, for example, in annual killifish (oviparous fish from the suborder Aplocheiloidei) which inhabit temporary ponds of Africa and South America [23,24]. These ponds dry out seasonally, killing all adults and juveniles. Only embryos buried in the soil persist until the next rainy season. Diapause in annual killifish can occur in three developmental stages [25,26]. Diapause I occurs between epiboly and the onset of embryogenesis, in a dispersed blastomere stage specific to annual fish, diapause II occurs in the long-somite embryo before most organs develop, and finally, diapause III occurs when the embryo is completely developed and ready to hatch. The presence of diapause I, II, and III and the different environmental cues that affect their onset, duration, and termination are highly variable among annual species [26-34]. Diapause can occur in supposedly optimal conditions for development, without apparent environmental stimuli, or it can be preceded by an environmental stimulus. In general, across annual species, diapause III is often obligatory whereas diapause I and II are often facultative. However, so-called escape eggs that do not seem to diapause can be present in clutches where other eggs do diapause (for example, [35], which used water and temperature change to provoke hatching), making it somewhat facultative at the individual level in many cases but constitutive at the level of a clutch.

Within the South American family *Rivulidae*, diapause occurs in all genera except in the former genus *Rivulus*[23,24,36,37]. These non-annual species commonly inhabit shallow areas of streams and swamps and their embryos generally develop in stable aquatic conditions [37]. Data of a few species suggest that they are continuous breeders [38,39]. It has been repeatedly suggested that diapause also occurs in some species of *Rivulus*[40,41], but the evidence is indirect and it remains to be verified in the controlled and standardized conditions used, for example, by Wourms [25,26]. Phylogenetic studies have not been conclusive on whether diapause might be the ancestral state for all Rivulidae and lost (perhaps several times) in non-annual *Rivulus*, or whether it was independently gained in the different annual groups [42-45]. A role of plasticity and genetic assimilation in the evolution of diapause has never been experimentally investigated, while it was already suggested in the scenarios for diapause evolution proposed by Wourms [26]. If genetic assimilation has played a role in the evolution of diapause in this group, a plastic developmental arrest provoked by an unusual environment should be the ancestral condition relative to the genera with annual diapausing species. Diapause is a type of dormancy where adverse environmental conditions are not immediately halting development or where dormancy occurs without adverse conditions [46]. Therefore, the ancestral plastic developmental arrest could have been a form of quiescence, an immediate response to adversity which subsequently became lagged and constitutive, or a plastic diapause with a lagged effect from the start. The developmental arrest might not have been complete from the start either, being just a slowing down of development. Determining where exactly in the phylogeny diapause originated then becomes conditional on the environments in which diapause is assessed. It then seems better not to see diapause anymore as a presence-absence trait but as a probabilistic trait, determined by a liability to diapause in a given environment.

To investigate a possible role of plasticity and genetic assimilation in the evolution of diapause, we analyzed plasticity in developmental timing in embryos of five species of the former non-annual killifish genus *Rivulus*, limited to one pair per species*.* Desiccation might provide an extreme environment and a cue rarely occurring for non-annual killifish. It is probably much more common in the environments where annual species evolved, making it a relevant candidate cue to investigate. If desiccation would occur at ages and in a stage where diapause III normally takes place, hatching might become impossible or non-observable because of lack of water. We therefore applied desiccation at very young ages. We exposed embryos to brief periods of desiccation and estimated developmental rates and survival during and after this treatment. If a plastic response occurs during desiccation towards a slower development, this would argue for plastic desiccation quiescence being the ancestral condition for diapause in a given stage. If, on the other hand, we observe delayed responses to desiccation, this would support the possibility of a plastic diapause being the ancestral condition.

## Methods

### Housing, data collection, and development monitoring

The five pairs used in this study were of the following species, all belonging to the former genus *Rivulus* before a recent taxonomic revision [37]: *Cynodonichthys brunneus*, *Cynodonichthys magdalenae*, *Cynodonichthys kuelpmanni*, *Anablepsoides immaculatus*, and *Laimosemion frenatus*. All five pairs were kept in 20-L aquaria in a climate room in 12-hour light/dark cycle and at 19°C except for the *C. magdalenae* pair, which was kept at 23°C with an aquarium heater. Each individual aquarium contained a floating acrylic wool mop, which served as a substrate where the fish deposited their eggs.

The day before collecting eggs, a clean mop was introduced in the aquarium. After 24 hours the mop was removed, eggs were collected and placed individually in wells of a 24-well plate with 1 mL of sterilized water. In a pilot aimed to assess egg numbers and developmental times, the first 62 embryos (10 *L. frenatus* and 52 *C. brunneus* embryos) were kept in this sterilized water for the entire duration of the experiment (Test group). We imposed the desiccation treatment on approximately half of the other eggs, always 24 hours after collection each collected clutch was divided in two equal groups if possible or nearly so. For half of the embryos, the water was removed from the well and plates were placed in desiccators with demineralized water saturating the air at 100% relative humidity (Desiccation group). This constant humidity level was verified with a datalogger. The other half of the eggs were kept in the original sterilized water (Control group). The desiccation treatment lasted 2 weeks for some embryos but in order to save more eggs for observation after the treatment, desiccation was limited to approximately 1 week thereafter. After the treatment period, 1 mL of sterilized water was again added to the well. We collected eggs during approximately 4 months and obtained a total of 383 embryos.

Based on previous studies in killifish [25], development was divided in five stages that can be distinguished well. Briefly, embryos were assigned to stage one from the appearance of the perivitelline space. A neural keel and the first somites appearing delineate the start of stage two, the presence of optic cups the transition to stage three and the pigmentation of the eyes the transition to stage four. Finally, stage five starts when the embryo completely surrounds the yolk sac and extends until the fry hatches. Developmental stages were determined approximately every 2 days for each individual embryo and the desiccation treatment was ended on these days as well, causing differences between individuals in the exact duration of the treatment. For embryos in stages four and five, we observed each time whether the tail coiled over the left or right side of the head. Many embryos hatched spontaneously in both treatments in the experiment and were further raised successfully. For a small number of embryos in stages four and five that did not hatch spontaneously, water with peat extract was added to the well to investigate whether this could provoke hatching, as it does in annual rivulid killifish (Van Dooren, personal observation).

### Statistical analysis

Developmental life histories consist of time-to-event data, durations which here were the age intervals embryos spend in each developmental stage before either moving into the next stage, hatching, or dying. Time-to-event data are typically analyzed with methods commonly known as ‘survival analysis’ that are designed to take censoring into account (an accessible reference is [47]). The main focus of this analysis is on modelling rates of moving between developmental stages (speeds of developmental processes) and on the mortality rates per stage, including an assessment of their dependence on explanatory variables. The data were therefore analyzed using Cox’s proportional hazard modeling, using library survival in R [48,49]. We modeled all transition rates separately, that is, the mortality rates per stage, and per stage the developmental transitions to the next stage. When considering a mortality rate for individuals at risk of dying in a certain stage, all other transitions than death are treated as censors, causing the individual to be not at risk anymore. The same holds for all developmental transitions, where deaths were treated as censors. Hatching from stage five was included as a developmental transition. Hatching of individuals after adding peat water, was treated as a censor at the moment of adding water, that is, their hatching was not considered to be an event in the model. We thus only analyzed rates of spontaneous hatching. In order to investigate immediate effects of experimental conditions and delayed responses, we investigated effects of the treatment group to which each egg belongs, whether the response when returned to water became different in this second period, and tested for effects of the total length of the dry period on embryos back in water. If only immediate responses to desiccation occur (quiescence), we expect no effect of the length of the dry period on developmental and survival rates after that period. In this case, we also expect that treatment effects disappear after eggs are returned to water, which would lead to a significant treatment:period interaction. If, on the other hand, only significant main treatment effects are found without interactions, treatments have the same effects throughout development and thus the desiccation treatment has persistent effects after embryos have been returned to water (diapause). In the maximal models fitted to each transition rate, effects of pair, period (first treatment conditions or wet again after dry period) treatment group (test, control, desiccation), total duration of the dry period experienced, and their interactions were present as fixed effects and we thus assessed whether each of these affected survival or development. Plate effects were added as a random frailty effect. Model simplification in order to remove non-significant effects was carried out using likelihood ratio tests. The plate effect was tested for significance first. Then non-significant interactions and subsequently non-significant main effects were removed from the maximal models. Stage-specific mortalities were analyzed with a dependence of the baseline hazard on age. For developmental transitions we used time since entry in that stage instead, as time-in-stage seems a more natural time scale here. To understand significant pair/treatment or pair/period interactions, we inspected pairwise contrasts and their family-wise simultaneous confidence intervals [50] and indicate which pairs are significantly different. For the control groups, we looked at differences in rates between pairs. For the treatment groups or for the second period, we looked at whether the pair-specific treatment effects on these rates were different from zero or not.

We made overview figures of the main results using routines in the mstate library [51]. We plot the probabilities of being in either of the developmental stages or dead as a function of age. Individuals that hatched spontaneously were assumed to stay alive, to bring out more clearly in the figure which proportion of embryos hatched asymptotically. In order to investigate whether our experimental treatment in the end affected ages at hatching, we fitted parametric survival regression models (function survreg ()) with a log-logistic or lognormal distribution, pair and collection day effects and their interactions, and tested for an overall effect of the experimental treatment or an interaction with pair effects using likelihood ratio tests.

We used the frequency of switches in the coiling direction of the tail as a measure of individual activity, for stage four and five embryos only. We analyzed per stage whether the frequency of individual changes in the coiling direction of the tail changed with the amount of time spent in that stage, whether there were treatment effects and pair effects (and their interactions). A change since the previous observation was coded as positive response, if the embryo coiled the tail on the same side of the head this was recorded as a negative response. Generalized linear models were used to analyze these data, in the family of binomial distributions. We used the cloglog link and the logarithm of the time interval since the previous observation as offset, so that the models, given a zero offset value, predicted the probability to change direction per day. As above, the initial maximal model included all explanatory variables and their interactions, and we then simplified using likelihood ratio tests. Note that the negative responses also include all embryos that changed coiling an even number of times since the last observation, the positive responses all embryos with uneven numbers of changes.

The probabilities of hatching when water with peat extract was added are reported.

## Results

**Figure 1:**
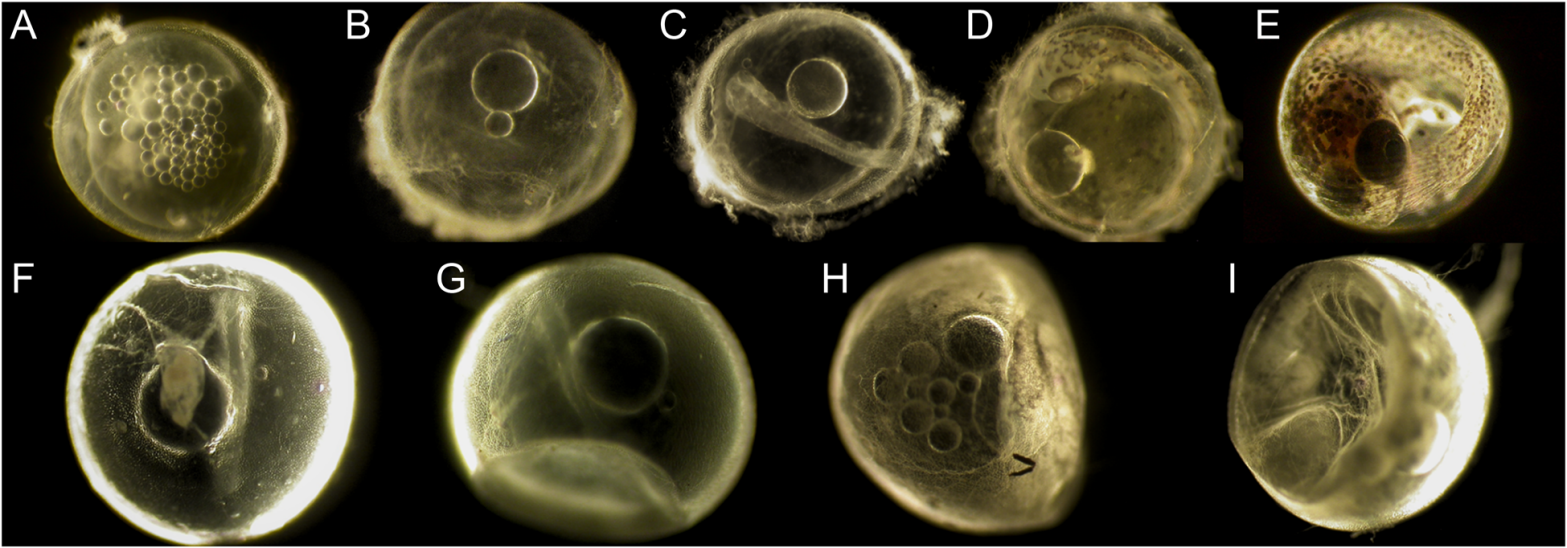
**Upper row: developmental stages in *Rivulus *spp. (A)** Stage one: approximately 50% epiboly; **(B)** Stage two: neural keel. **(C)** Stage three: optic cups; **(D)** Stage four: pigmentation in eyes and skin; **(E)** Stage five: embryo surrounding yolk sac. Lower row: **(F-I)** Morphological effects of air-exposed development. **(A, B, F, G)***C. magdalenae*. **(C, H, I)***C. brunneus*. **(D)***L. frenatus*. **(E)***C. kuelpmannii*. Images are not to scale.

We collected 209 eggs from *C. brunneus*, 112 from *C. magdalenae*, 23 from *C. kuelpmanni*, 11 from *A. immaculatus*, and 10 eggs from *L. frenatus* (all 10 in the test group). Numbers of embryos that reached each developmental stage and numbers hatched are given per pair and per treatment in Table 1. The overall median time duration of the desiccation treatment among the embryos that were returned to water was 8 days (eight for *C. brunneus*, six for *C. magdalenae*). Effects of developing in air were visible few hours after the start of the treatment. Generally, after 48 hours egg envelopes became opaque and rough, depressions in the egg surface appeared and eggs started shrinking (Figure 1). However, these effects were variable in different embryos (see Figure 1I and H for embryos from the same clutch experiencing identical conditions). Few embryos were returned from the desiccation treatment into water before they reached stage three. Therefore, assuming a potential lack of power, we did not expect any significant period effects and effects of the duration of the dry period experienced for stages one and two. In none of the analysis, rates in the Test and Control groups were different and we pooled these groups for the presentation of results. We therefore only present Control and Desiccation treatment levels.

**Table 1:** Number of embryos observed per pair, treatment, and developmental stage

**Pair/Species**	**Stage 1**	**Stage 2**	**Stage 3**	**Stage 4**	**Stage 5**	**Hatched**
*C. brunneus*	133/76	112/55	112/35	110/22	109/15	109/15
*L. frenatus*	10	10	8	7	6	6
*C. kuelpmanni*	11/12	6/11	6/11	6/9	5/0	5/0
*A. immaculatus*	5/6	4/4	4/4	4/3	4/0	4/0
*C. magdalenae*	52/60	47/54	46/53	46/46	26/34	24/29

Numbers before the slash are for the Control, after the slash for the Desiccation treatment. In the Hatched column, embryos that hatched in response to a water change are included.

### Survival

**Figure 2:**
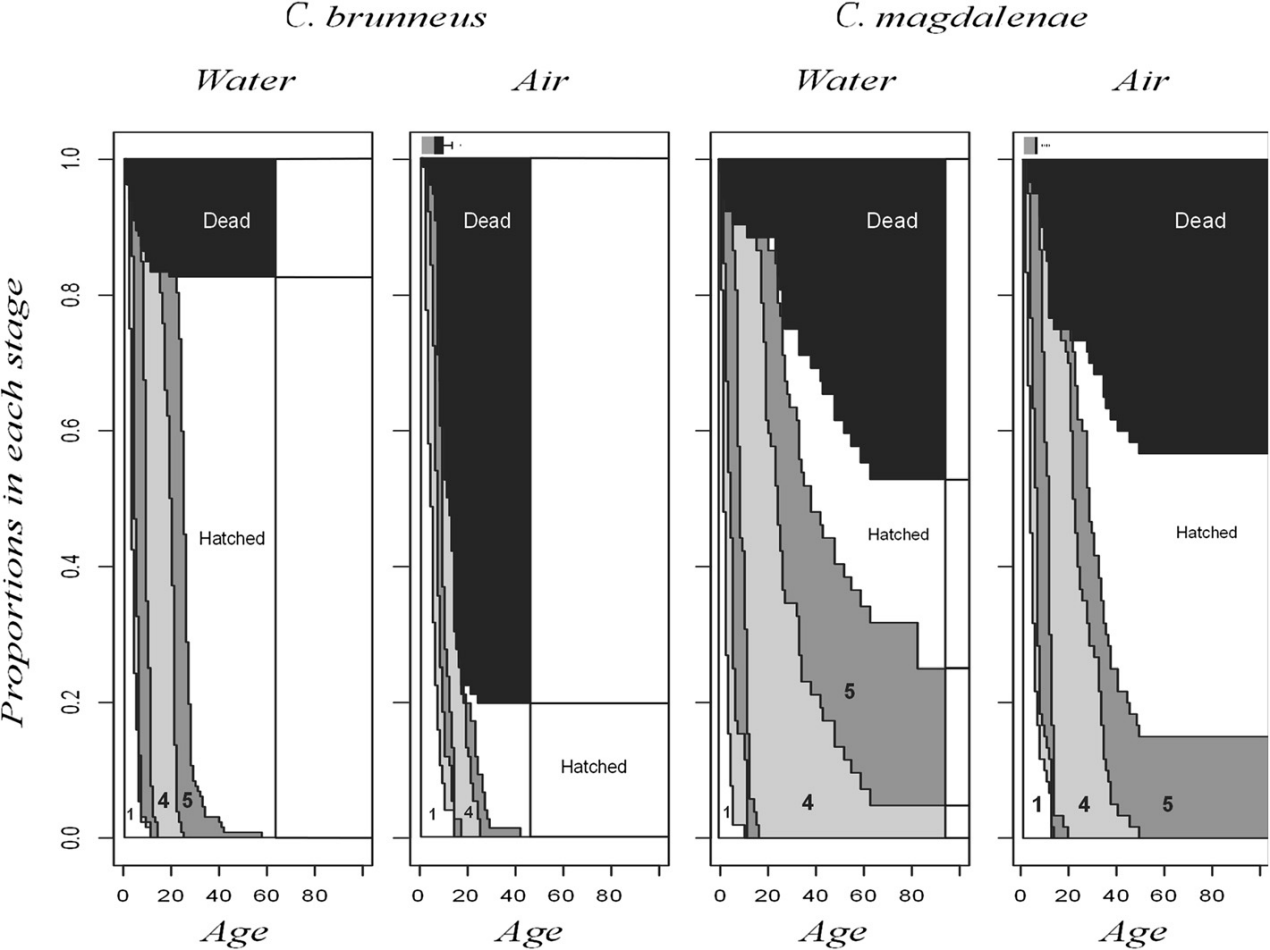
**Probabilities for being in each stage in dependence on age.** All individuals start in developmental stage one. Developmental stages are labeled where possible. From left to right, one can track the bands for successive stages where adjacent stages always have different gray levels. Only data from two pairs are represented, the ones which have the largest sample sizes. There are two absorbing states: death (black) and hatched (white), where we assume that hatched individuals do not die within this experiment such that proportions hatched and dead at the end of the experiment indicate actual proportions of individuals in each of these absorbing states. The ‘water’ groups consist of individuals in the two different control groups pooled. *C. brunneus* embryos were observed for a shorter duration than *C. magdalenae*, and per panel, we stop the shading at the oldest age where embryos were observed. Gray bars above the ‘Air’ panels for the Desiccation groups indicate the ages where all embryos were in the treatment. Black box plots to the right of these bars indicate the distribution of lengths of dry periods experienced by surviving individuals. It can be seen that there is more variation in the length of the dry period experienced for *C. brunneus* embryos but that minimum periods are very similar between pairs.

We found no immediate nor delayed effects of the experimental treatment on survival in stages one, two, and five (Table 2, Figure 2). Desiccation significantly increased mortality overall in embryos in stages three and four (Table 2, Figure 2). We found a significant period:pair interaction on mortalities in stage four (Table 2) due to the fact that in comparison to embryos of the other pairs, mortality was reduced for embryos of the *C. magdalenae* pair when they were returned to water after the desiccation treatment (Table 3). Thus, the desiccation treatment has persisting survival effects in all pairs, however only for stage three in the embryos of *C. magdalenae*. Individual differences in the total duration of the dry period experienced did not affect survival when returned to water significantly.

**Table 2:** Period and desiccation effects on survival and development, by stage

**Mortality rates - age-dependent baseline hazard**				
**Stage**	**Period (Coef. (S.E.))**	**LTR**	**Desiccation treatment (Coef. (S.E.))**	**LTR**
1		NS		NS
2		NS		NS
3		NS	2.79 (0.78)	Chisq = 13.25, df = 1, p = 0.0003
4	Interaction	Chisq =27.60, df = 3, *P* <0.0001	4.74 (1.16)	Chisq = 12.02, df = 1, p = 0.0005
5		NS		NS
**Developmental rates - baseline hazard depends on time since entry in each stage**				
**Stage**	**Period (Coef. (S.E.))**	**LRT**	**Desiccation treatment (Coef .(S.E.))**	**LRT**
1		NS	-0.61 (0.14)	Chisq = 33.5, df = 4.83, p < 0.0001
2	When back in water - 1.61 (1.07)	Chisq =5.96, df = 1.68, *P* = 0.037	Interaction	Chisq = 21.73, df = 1.5, p < 0.0001
3	Interaction	Chisq = 24.79, df = 4, *P* <0.0001	-0.93 (0.25)	Chisq = 19.05 df = 1, p < 0.0001
4		NS	Interaction	Chisq = 14.72, df = 1, p = 0.0001
5		NS		NS
**Hatching**		NS		NS

A positive coefficient of an effect implies increased mortality or developmental rate, whereas negative coefficients indicate the opposite. The chi-square value (Chisq), degrees of freedom (df), and tail probabilities are given for each significant effect. Interactions of a treatment effect and pairs are indicated by ‘INTERACTION’ and these are further elaborated in Tables 3 and 4. For development in stage three, we also found an effect of the total number of days experienced in dry conditions. It reduces the development rate into stage four of embryos of pair one when returned to water by -13.157 (0.287) per day previously spent in dry conditions. The decrease in log-likelihood when this effect is added to the model is 13.77. NS indicates non-significant effects.

**Table 3:** Interactions of experimental conditions (Period effect, during and after the desiccation treatment) with pair effects on mortality rates

**Stage**	**4**	
**Pair/Species**	**Control**	**Change after return to water (Period effect)**
*C. brunneus*	Baseline	-3.92 -1.44
*L. frenatus*	-1.45 -5.74	NA
*C. kuelpmanni*	-4.07 -2.88	-2.61 -4.10
*A. immaculatus*	NA (Wide)	NA (Wide)
*C. magdalenae*	-1.27 -3.97	-7.53 - -1.07*

Confidence intervals are given. One interval that does not include zero is indicated by ‘*’. Embryos of that pair have a significantly reduced mortality after return to water. In the ‘NA’ cases, the confidence intervals were estimable but extremely wide.

### Developmental rates

We found no effects of the experimental treatments or pair effects on the rate of hatching from stage five (Table 2). Desiccation had a negative effect on developmental rates in stages one to three (Tables 2 and 4). Decreased developmental rates occurred across all pairs in stage one, but are pair-specific in the other stages. In stages two and three, the effects of desiccation changed when embryos were returned to water. In stage two, only the *C. magdalenae* embryos had no decreased developmental rate during the dry period (Table 4), but unexpectedly pairs did have an overall reduced rate of development when back in water (period effect in stage two, Table 2). In stage three, all pairs had reduced developmental rates during the dry period. The embryos of the *C. brunneus* pair seemingly developed much faster when returned to water (Tables 2 and 4), but when we combine the significant negative effect of the total number of days experienced in dry conditions (average 8 days in *C. brunneus* embryos) with the overall rate increase, then there is only faster development for embryos that experienced the shortest dry periods and a decrease when they experienced a larger than average number of dry days.

**Table 4:** Interactions of experimental conditions with pair effects on developmental rates

**Stage**	**1**	**2**		**3**		**4**	
	**Control**	**Control**	**Desiccation treatment**	**Control**	**Effect post desiccation**	**Control**	**Desiccation treatment**
*C. brunneus*	Baseline	Baseline	-2.61 - -0.95*	Baseline	75.12 - 84.39*	Baseline	-1.55 - 0.10
*L. frenatus*	-1.56 - 0.69	-2.22 - 1.27	NA	-2.82 - 2.10	NA	-2.20 - 0.27	NA
*C. kuelpmanni*	-0.16 - 1.92	-0.05 - 3.22	-4.50 - -0.76*	-1.58 - 1.18	NA	-3.61 - -0.56*	NA
*A. immaculatus*	0.34-3.30*	-0.30 - 3.70	-4.82- -0.27*	-1.35 - 2.59	NA	-0.43 - 2.71	NA
*C. magdalenae*	0.37 - 1.62*	-1.48 - 0.36	-0.75 - 0.89	-1.46 - 0.50	-7.05 - 2.77	-2.88 - -1.35*	-0.14 - 1.32

Confidence intervals are given. Intervals that do not include zero are indicated by “*”. The sign of their limits indicates a negative or positive effect on a developmental rate. The pair effect on development in stage one is significant, but there is no interaction with experimental treatments (Chisq = 45.61, df = 7.32 *P* <0.0001). It is added for completeness.

In stage four, the embryos of the *C. magdalenae* and the *C. kuelpmanni* pairs generally developed slower than the other pairs in the control treatment. Note that embryos of *C. magdalenae* remained in stages four and five for prolonged amounts of time, even in the absence of the desiccation treatment and that embryos that were exposed to desiccation stop hatching at an age of about 50 days and with negligible further mortality (Figure 2). In the control treatment, the stage four embryos showed no developmental transitions from the age of 50 days on, since there were no further events in this group (approximately 25% of unhatched embryos). In the desiccation treatment, this was the case for the stage five embryos (100% of unhatched embryos). Three of the embryos from the *C. kuelpmanni* pair also remained in stage five for over 30 days, indicating that the arrest we observed might not be specific to one particular pair or species or an effect of the temperature at which the adults were kept.

### Coiling direction

At all times, about half of the embryos had their tails coiled over the left side of the head. We only analyzed changes in switching frequency for *C. brunneus* and *C. magdalenae* embryos and report predictions from the model obtained after model selection at three times within stage, which can be beyond the actual range observed. For the stage four embryos, the frequency of switching coiling direction did not depend on treatment. Among the *C. brunneus* pair embryos*,* the frequency of switching did not decrease with time spent in that stage and remained at 27.5% (s.e. 2.1). In *C. magdalenae*, switching frequency declines with time spent in stage four from 29.1% (s.e. 7.5) at zero days in stage four to 0.1% (s.e. 0.2) at day 30 (pair:age interaction chisq = 10.65 df = 1 *P* = 0.001). Among the stage five embryos, the frequency of switching coiling direction did not depend on treatment. There was a significant difference in initial frequency between pairs (chisq = 42.91 df = 1 *P* <0.001). In both pairs switching frequency declined significantly with time spent in stage five (chisq = 28.39 df = 1 *P* <0.001). The decline did not have different slopes for both pairs. In *C. brunneus* switching frequency is predicted to decline with time spent in stage five from 28.9% (s.e. 2.3) at zero days in stage five to 11.2% (s.e. 4.8) at day 30 and 4.1% (s.e. 3.8) at day 60. In *C. magdalenae*, the predictions for switching frequency are 15.7% (s.e. 2.1) at zero days in stage five to 5.5% (s.e. 0.9) at day 30 and 1.8% (s.e. 0.7) at day 60.

### Hatching

We did not find treatment effects on the ages at which embryos actually hatched spontaneously, either modelling hatching times with log-normal or log-logistic distributions. In the *C. brunneus* embryos, this can be partially explained by the significant compensatory response in stage three when embryos are returned to water. For the embryos of the *C. magdalenae* pair, there are no significant compensatory responses, but Figure 2 and Table 4 suggest that they might occur in stage four.

Water with peat extract was added to three *C. magdalenae* embryos that were in stage four for over 30 days. One of these hatched. This suggests that the embryos of the *C. magdalenae* pair that arrested development in what we scored as stage four, were in fact completely developed and simply relatively small. The addition of water with peat extract provoked hatching in stage five embryos as well. We added water to nine *C. magdalenae* embryos on a first day and to nine different embryos 2 weeks later. The minimum time by then spent by these embryos in stage five was 19 days. Among these of the first day, seven hatched and eight among those of the second day. From these stage five embryos, five were in the Desiccation treatment and had already been for over 30 days in stage five. Three of them hatched, demonstrating that the arrest we observe is not a dead end and that alevins can continue their life cycle. Sample sizes of embryos per age and treatment group were small and make the power of any statistical analysis low.

## Discussion

Our analysis of developmental and hatching rates shows that developmental rates in the *Rivulus* embryos we investigated are plastic when they are exposed to desiccation. The embryos usually decrease the speed at which they develop. In developmental stages one to three, this occurs during the treatment and when the embryos were returned to water, such that the response observed is not simply in the direction of quiescence in response to adverse conditions but involves effects that are delayed relative to the presence of the desiccation cue, as in diapause. However, there is no plastic arrest or diapause in these stages, only a general slowing down of development. There is one exception to this decrease in developmental rates, namely embryos of the *C. brunneus* pair that experience the shortest dry periods in this experiment develop faster from stage three to stage four after being returned to water, which compensates for the loss in total developmental time. Unexpectedly, age at hatching is not affected by this plasticity.

We have found that about 25% of the stage four *C. magdalenae* Control embryos alive at the end of the experiment were already in that stage for over 60 days and that neither mortality nor hatching had occurred for at least 30 days. It was found that these embryos are capable of hatching such that we conclude that they are in fact completely developed but relatively small individuals. A brief period of desiccation increased the cohort of *C. magdalenae* embryos without mortality or hatching in the preceding month to 100% of the unhatched embryos at the end of the experiment. This arrest seems plastic: either the proportion of such embryos increases with desiccation, or alternatively more embryos enter the arrest at earlier ages in desiccation conditions. Part of the stage five embryos in the Control group might be in a delaying stage as well but not easy to detect as such. In this group, there are stage five *C. magdalenae* embryos hatching until the end of the experiment, so that certainly not the full 100% of individuals have arrested development at that point. However, hatching might still stop when embryos become older than about 80 days, but we did not extend our observations much further than that age.

Diapause differs from quiescence in that it is not an immediate response to the environment and it can even occur in the absence of adverse environmental conditions [46,52]. To exclude as much as possible cases where hatching is delayed because of physiological malfunction, Wourms [26] characterized diapause III in stage five annual killifish embryos using seven criteria. Wourms [26] required that diapause should similarly occur in all embryos of an egg population, precluding the detection of facultative diapause where fractions of embryos diapause. We therefore propose not to use this criterion further. We did not measure yolk reserves, as this seemed impossible to carry out in a non-invasive manner. We observed the absence of developmental transitions or hatching in two cohorts for over 30 days. The presence of such a developmental arrest is a minimal requirement for diapause. Three criteria aim to detect a decrease in embryonic and metabolic activity and are therefore useful in differentiating diapause from delayed hatching without decreased activity [30,53-55]. Instead of scoring spontaneous motility or cardiac activity at the moment of observation, we looked at coiling direction of the tail relative to the head, which is an integrative measure of activity during the period preceding the observation. Our analysis of coiling direction demonstrates that the frequency of switching direction can be pair or species-specific, that it decreases over time and that there is no effect of desiccation. We therefore observe a decrease in general activity during late developmental stages. We could handle plates with embryos repeatedly without provoking hatching, in agreement with the requirement that casual interference should not provoke hatching. When embryos delay hatching without lowering metabolism and activity, prolonged delays will lead to high mortality [53,56]. Therefore, the last criterion is that there should be no deleterious effects of prolonged arrest. Mortality in the period where we observed a developmental arrest was negligible. We conclude that the arrest we observed in pre-hatching stages in the embryos of one pair of *C. magdalenae* could correspond to diapause III. Although direct and repeated measures of metabolic activity in these embryos are lacking, the length of the non-deleterious arrest in these embryos suggest that a certain depression in activity should be present. However, a more extensive analysis of metabolic activity would be needed to demonstrate it. We note that among the annual rivulids, data on metabolic activity, embryo developmental rates, and survival are limited to a small set of species. A study on *Austrofundulus limnaeus*[55] considers embryos to be in diapause III when metabolic activity decreases, not when it is below a certain level. In our opinion, analyses where rates of development and survival are assessed over prolonged periods of time should be carried out for many more annual species. Some studies on annuals do report the probabilities of being in a number of stages across time [32], which make it easier to spot the absence of development and mortality.

Survival in stages one and two, stages in which embryos were generally in different treatment, was not affected by desiccation. Developing in air immediately reduced the survival of *Rivulus* embryos in stages three and four. In the *C. magdalenae* pair and in stage four, survival increased again when the embryos were returned to water. In the other pairs, survival effects of desiccation persisted when embryos were rewetted. The effect does not seem to have become constitutive, given that there are no differences remaining in survival once the embryos reached stage five. The length of the desiccation period did not have an effect on survival, suggesting that the increased mortality in later stages is not due to a frailty that gradually accumulated during the time the embryo spent in the treatment. Further development and successful hatching were also not compromised by a period of desiccation during early development. Embryos still hatch spontaneously in the Desiccation treatment, embryos already for longer periods in a late developmental stage can respond to a hatching stimulus, and making the duration of the dry period longer only has a significant effect on developing from stage three to four. If there is any persistent physiological malfunction due to the brief period of desiccation, it seems limited.

Our results demonstrate that the hypothesis of a plastic developmental delay or diapause being the ancestral condition for the family *Rivulidae* followed by genetic assimilation modifying the degree of plasticity in the annual groups is worth a detailed investigation on a large group of annual and non-annual rivulid species where experiments of the kind we present here are used to assess developmental plasticity. These experiments should include several breeding individuals per species in order to separate between from within-species variation, which was impossible in this study. A more extensive analysis should include some non-rivulid killifish as well. We have shown developmental plasticity in the direction of typical patterns of annual species in developmental stages one to three and five, but we also show that developmental arrest at pre-hatching stages, resembling annual diapause III, can occur in normal environmental conditions and that a compensatory acceleration of development can also take place. Given that Wourms [26] briefly reported induction of diapause I and II in response to desiccation in several species of annual killifish, where diapause III is generally obligatory, it seems that the differences between non-annual and annual species will indeed be quantitative rather than qualitative. The fact that an arrest such as diapause III has to be scored using a combination of individual and cohort criteria, indicates that obtaining reliable and repeatable individual quantitative measures of diapause is not easy and requires further work.

Developmental plasticity in response to desiccation has already been found in the marine mummichog *Fundulus heteroclitus* belonging to a different killifish family without annual species [57]. Its embryos can develop aerially between spring tides in marshes. *F. heteroclitus* embryos accelerate development when incubated in air, but can only hatch when water is present [57]. Desiccation provokes a rapid response at the transcriptome level which includes a sensing stage triggering the activation of signals and mechanisms to cope with desiccation, followed by changes in metabolism and morphogenesis responsible for the developmental acceleration [58]. Although extremely valid, transcriptome changes can currently not be used as qualitative measures of individual diapause in an experiment such as ours, as the technique does not allow non-invasive monitoring. When RNA from different individuals in a group is pooled for analysis [58], the results are not suited to investigate inter-individual variability given the same environmental conditions, which will be essential to understand diapause evolution. Desiccation up to 12 days apparently did not affect survival of *F. heteroclitus* embryos irrespective of their developmental stage, demonstrating a substantial desiccation resistance in this non-annual species. Annual embryos are relatively resistant to desiccation throughout development but diapause II has been shown to be the most resistant stage in embryos of the South-American *Austrofundulus limnaeus*[59] and the non-rivulid African *Nothobranchius guentheri*[31]. We found that in *Rivulus* survival is most affected by desiccation in stages three and four, with diapause II expected in rivulids at the end of stage two or the start of stage three. This implies that if diapause II would occur here, it would not be in a stage very resistant to desiccation. The studies beyond the rivulids underline that desiccation responses in killifish are a dynamic process on individual and evolutionary time scales, warranting further within- and between-species comparisons of rates of development and of stage-specific mortalities.

Other cues than desiccation might be involved in plasticity evolution and genetic assimilation processes of the different diapauses. Annual fish embryos have been shown to be specifically tolerant to different stressors including desiccation, anoxia, and osmotic stress [55,59-62]. Next to experimental comparative studies, we should therefore obtain more data on multivariate environmental conditions experienced by annuals and non-annuals in the field, with the aim of reconstructing which conditions were rare and common for the ancestral species. Annual killifish development differs from the common teleost development in the occurrence between epiboly and the onset of organogenesis of the dispersed cell stage in which the blastomeres individually disperse over the segmentation cavity and where diapause I can occur [63,64]. It has been suggested that this stage is relatively insensitive to hypoxia [63]. It thus seems relevant to investigate whether aspects of this developmental process depend on oxygen availability in both annual and non-annual rivulids.

## Conclusions

We have shown that embryos of non-annual *Rivulus* species can respond to desiccation by decreasing their developmental rates in early development. This effect persisted after they were returned to water, which argues for a response in the direction of diapause as opposed to quiescence. In later developmental stages, we found a plastic arrest resembling diapause III in the embryos of the *C. magdalenae* pair. This arrest is present in control conditions and increases in frequency in the desiccation treatment. These results suggest that plastic developmental delays or diapauses could be the ancestral state for the *Rivulidae* family and are in agreement with the hypothesis that the evolution of plasticity played a role in their emergence. Further comparative experimental studies on a large group of annual and non-annual species and field research are needed to better understand how diapause evolved in this group.

## Competing interests

The authors declare no competing interests.

## Authors’ contributions

TJMVD conceived the study. IVL and TJMVD performed the experiments, analyzed the data, and wrote the manuscript. Both authors read and approved the final manuscript.

## References

[B1] Woltereck R Weitere experimentelle Untersuchungen über Artveränderung, speziel über das Wesen quantitativer Artunterschiede bei Daphniden Ver dtsch zool Gesell 1909 19 110 173

[B2] Scheiner SM Genetics and evolution of phenotypic plasticity Annu Rev Ecol Sist 1993 24 35 68 10.1146/annurev.es.24.110193.000343

[B3] Philippi T Seger J Hedging one’s evolutionary bets, revisited Trends Ecol Evol 1989 4 41 44 10.1016/0169-5347(89)90138-9 21227310

[B4] Dempster ER Maintenance of genetic heterogeneity Cold Spring Harb Symp Quant Biol 1955 20 25 32 10.1101/SQB.1955.020.01.005 13433552

[B5] Leimar O Hammerstein P Van Dooren TJM A new perspective on developmental plasticity and the principles of adaptive morph determination Am Nat 2006 167 367 376 10.1086/499566 16673345

[B6] DeWitt TJ Sih A Wilson DS Costs and limits of phenotypic plasticity Trends Ecol Evol 1998 13 77 81 10.1016/S0169-5347(97)01274-3 21238209

[B7] Auld JR Agrawal AA Relyea R Re-evaluating the costs and limits of adaptive phenotypic plasticity Proc Biol Sci 2010 277 503 511 10.1098/rspb.2009.1355 19846457 PMC2842679

[B8] Waddington CH Canalization of development and the inheritance of acquired characters Nature 1942 150 563 565 10.1038/150563a0 13666847

[B9] Lande R Adaptation to an extraordinary environment by evolution of phenotypic plasticity and genetic assimilation J Evol Biol 2009 22 1435 1446 10.1111/j.1420-9101.2009.01754.x 19467134

[B10] Pigliucci M Murren CJ Perspective: genetic assimilation and a possible evolutionary paradox: can macroevolution sometimes be so fast as to pass us by? Evolution 2003 57 1455 1464 10.1111/j.0014-3820.2003.tb00354.x 12940351

[B11] West-Eberhard MJ Developmental Plasticity and Evolution 2003 Oxford: Oxford University Press

[B12] Pfennig DW Wund MA Snell-Rood EC Cruickshank T Schlichting CD Moczek AP Phenotypic plasticity’s impacts on diversification and speciation Trends Ecol Evol 2010 25 459 467 10.1016/j.tree.2010.05.006 20557976

[B13] Scheiner SM Selection experiments and the study of phenotypic plasticity J Evol Biol 2002 15 889 898 10.1046/j.1420-9101.2002.00468.x

[B14] Palmer AR Symmetry breaking and the evolution of development Science 2004 306 828 833 10.1126/science.1103707 15514148

[B15] Wund MA Valena S Wood S Baker JA Ancestral plasticity and allometry in threespine stickleback fish reveal phenotypes associated with derived, freshwater ecotypes Biol J Linn Soc Lond 2012 105 573 583 10.1111/j.1095-8312.2011.01815.x 22611287 PMC3351840

[B16] Losos JB Creer DA Glossip D Goellner R Hampton A Roberts G Haskell N Taylor P Ettling J Evolutionary implications of phenotypic plasticity in the hindlimb of the lizard Anolis sagrei Evolution 2000 54 301 305 10937208 10.1111/j.0014-3820.2000.tb00032.x

[B17] Chapman LG Galis F Shinn J Phenotypic plasticity and the possible role of genetic assimilation: Hypoxia-induced trade-offs in the morphological traits of an African cichlid Ecol Lett 2000 3 387 393 10.1046/j.1461-0248.2000.00160.x

[B18] Ledon-Rettig CC Pfennig DW Nascone-Yoder N Ancestral variation and the potential for genetic accommodation in larval amphibians: implications for the evolution of novel feeding strategies Evol Dev 2008 10 316 325 10.1111/j.1525-142X.2008.00240.x 18460093

[B19] Gomez-Mestre I Buchholz DR Developmental plasticity mirrors differences among taxa in spadefoot toads linking plasticity and diversity Proc Natl Acad Sci U S A 2006 103 19021 19026 10.1073/pnas.0603562103 17135355 PMC1748170

[B20] Aubret F Shine R Genetic assimilation and the postcolonization erosion of phenotypic plasticity in island tiger snakes Curr Biol 2009 19 1932 1936 10.1016/j.cub.2009.09.061 19879141

[B21] Aubret F Bonnet X Shine R The role of adaptive plasticity in a major evolutionary transition: early aquatic experience affects locomotor performance of terrestrial snakes Funct Ecol 2007 21 1154 1161 10.1111/j.1365-2435.2007.01310.x

[B22] Spicer JI Burggren WW Development of physiological regulatory systems: altering the timing of crucial events Zoology 2003 106 91 99 10.1078/0944-2006-00103 16351894

[B23] Myers GS Annual fishes Aquar 1952 23 125 141

[B24] Myers GS Studies on South American freshwater fishes I Stanford Ichthyol Bull 1942 2 89 114

[B25] Wourms JP Developmental biology of annual fishes. I. Stages in the normal development of Austrofundulus myersi Dahl J Exp Zool 1972 182 143 167 10.1002/jez.1401820202 5079083

[B26] Wourms JPP The developmental biology of annual fishes. III. Pre-embryonic and embryonic diapause of variable duration in the eggs of annual fishes J Exp Zool 1972 182 389 414 10.1002/jez.1401820310 4674089

[B27] Inglima K Perlmutter A Markofsky J Reversible stage-specific embryonic inhibition mediated by the presence of adults in the annual fish Nothobranchius guentheri J Exp Zool 1981 215 23 33 10.1002/jez.1402150104 7229598

[B28] Matias JR Markofsky J The survival of embryos of the annual fish, Nothobranchius guentheri exposed to temperature extremes and the subsequent effects on embryonic diapause J Exp Zool 1978 204 219 228 10.1002/jez.1402040209

[B29] Markofsky J Matias JR The effects of temperature and season of collection on the onset and duration of diapause in embryos of the annual fish Nothobranchius guentheri J Exp Zool 1977 202 49 56 10.1002/jez.1402020107 925663

[B30] Levels PJ Gubbels RE Denucé JM Oxygen consumption during embryonic development of the annual fish Nothobranchius korthausae with special reference to diapause Comp Biochem Physiol A Comp Physiol 1986 84 767 770 10.1016/0300-9629(86)90403-2 2875847

[B31] Matias JR Embryonic diapause in annual fishes: evaporative water loss and survival Experientia 1982 38 1315 1317 10.1007/BF01954923

[B32] Levels PJ Denuce JM Intrinsic variability in the frequency of embryonic diapauses of the annual fish Nothobranchius korthausae, regulated by 1ight:dark cycle and temperature Environ Biol Fishes 1988 22 211 223 10.1007/BF00005382

[B33] Markofsky J Matias J Effects of light–dark cycles and temperature on embryonic diapause in the East African annual fish Nothobranchius guentheri Chronobiologia 1977 4 130 131

[B34] Markofsky J Matias JR Inglima K Vogelman J Orentreich N The variable effects of ambient and artificial light: dark cycles on embryonic diapause in a laboratory population of the annual fish Nothobranchius guentheri J Exp Biol 1979 83 203 215

[B35] Blažek R Polačik M Reichard M Rapid growth, early maturation and short generation time in African annual fishes Evodevo 2013 4 24 10.1186/2041-9139-4-24 24007640 PMC3844391

[B36] Huber JH Review of Rivulus, Ecobiogeography-Relationships 1992 Paris: Société Française d’Ichtyologie

[B37] Costa W Phylogenetic position and taxonomic status of Anablepsoides, Atlantirivulus, Cynodonichthys, Laimosemion and Melanorivulus (Cyprinodontiformes: Rivulidae) Ichthyol Explor Freshwaters 2011 22 233 249

[B38] Fraser DF Gilliam JF Nonlethal impacts of predator invasion: facultative suppression of growth and reproduction Ecology 1992 73 959 10.2307/1940172

[B39] Cassel M Mehanna M Mateus L Ferreira A Gametogenesis and reproductive cycle of Melanorivulus aff. punctatus (Boulenger, 1895) (Cyprinodontiformes, Rivulidae) in Chapada dos Guimarães, Mato Grosso, Brazil Neotrop Ichthyol 2013 11 179 192

[B40] Thomerson JE Taphorn DC Two new annual killifi shes from Amazonas territory, Venezuela (Cy pri nodontiformes: Rivulidae) Ichthyol Explor Freshwaters 1992 3 377 384

[B41] Costa W Relationships and taxonomy of the killifish genus Rivulus (Cyprinodontiformes: Aplo chei loidei: Rivulidae) from the Brazilian Amazonas river basin, with notes on historical ecology Aqua J Ichthyol Aquat Biol 2006 11 133 175

[B42] Parenti LR A phylogenetic analysis of Cyprinodontiform fishes (Teleostei; Atherinomorpha) Bull Am Mus Nat Hist 1981 168 335 557

[B43] Murphy WJ Collier GE A molecular phylogeny for aplocheiloid fishes (Atherinomorpha, Cyprinodontiformes): the role of vicariance and the origins of annualism Mol Biol Evol 1997 14 790 799 10.1093/oxfordjournals.molbev.a025819 9254916

[B44] Costa W Phylogeny and classification of Rivulidae revisited: evolution of annualism and miniaturization in rivulid fishes (Cyprinodontiformes: Aplocheiloidei) J Comp Biol 1990 3 33 92

[B45] Hrbek T Larson A The evolution of diapause in the killifish family Rivulidae (Atherinomorpha, Cyprinodontiformes): a molecular phylogenetic and biogeographic perspective Evolution 1999 53 1200 1216 10.2307/2640823 28565541

[B46] Danks HV Insect Dormancy: An Ecological Perspective 1987 Ottawa: Biological

[B47] Van Houwelingen H Modelling Survival Data in Medical Research, Volume 14 1995 London: Chapman & Hall

[B48] Therneau TM Grambsch PM Modeling Survival Data: Extending the Cox Model, Volume 20 2000 New York: Springer

[B49] R Development Core Team R: A Language and Environment for Statistical Computing 2008 Vienna: R Foundation for Statistical Computing

[B50] Hothorn T Bretz F Westfall P Simultaneous inference in general parametric models Biom J 2008 50 346 363 10.1002/bimj.200810425 18481363

[B51] De Wreede LC Fiocco M Putter H mstate: an R Package for the analysis of competing risks and multi-state models J Stat Softw 2011 38 1 30

[B52] Hand SC Podrabsky JE Bioenergetics of diapause and quiescence in aquatic animals Thermochim Acta 2000 349 31 42 10.1016/S0040-6031(99)00511-0

[B53] Darken RS Martin KL Fisher MC Metabolism during delayed hatching in terrestrial eggs of a marine fish, the grunion Leuresthes tenuis Physiol Zool 1998 71 400 406 10.1086/515423 9678500

[B54] DiMichele L Powers DA The relationship between oxygen consumption rate and hatching in Fundulus heteroclitus Physiol Zool 1984 57 46 51

[B55] Podrabsky JE Hand SC The bioenergetics of embryonic diapause in an annual killifish, austrofundulus limnaeus J Exp Biol 1999 202 2567 2580 10482717 10.1242/jeb.202.19.2567

[B56] Smyder EA Martin KLM Temperature effects on eggs survival and hatching during the extended incubation period of California Grunion, Leuresthes tenuis Copeia 2002 2 313 320

[B57] Tingaud-Sequeira A Zapater C Chauvigné F Otero D Cerdà J Adaptive plasticity of killifish (Fundulus heteroclitus) embryos: dehydration-stimulated development and differential aquaporin-3 expression Am J Physiol Regul Integr Comp Physiol 2009 296 R1041 R1052 10.1152/ajpregu.91002.2008 19193936

[B58] Tingaud-Sequeira A Lozano J-J Zapater C Otero D Kube M Reinhardt R Cerdà J A rapid transcriptome response is associated with desiccation resistance in aerially-exposed killifish embryos PLoS One 2013 8 e64410 10.1371/journal.pone.0064410 23741328 PMC3669298

[B59] Podrabsky JE Carpenter JF Hand SC Survival of water stress in annual fish embryos: dehydration avoidance and egg envelope amyloid fibers Am J Physiol Regul Integr Comp Physiol 2001 280 R123 R131 11124142 10.1152/ajpregu.2001.280.1.R123

[B60] Podrabsky JE Lopez JP Fan TWM Higashi R Somero GN Extreme anoxia tolerance in embryos of the annual killifish Austrofundulus limnaeus: insights from a metabolomics analysis J Exp Biol 2007 210 2253 2266 10.1242/jeb.005116 17575031

[B61] Machado BE Podrabsky JE Salinity tolerance in diapausing embryos of the annual killifish Austrofundulus limnaeus is supported by exceptionally low water and ion permeability J Comp Physiol B 2007 177 809 820 10.1007/s00360-007-0177-0 17581754

[B62] Podrabsky JE Hand SC Depression of protein synthesis during diapause in embryos of the annual killifish Austrofundulus limnaeus Physiol Biochem Zool 2000 73 799 808 10.1086/318106 11121353

[B63] Wourms JP The developmental biology of annual fishes. II. Naturally occurring dispersion and reaggregation of blastomeres during the development of annual fish eggs J Exp Zool 1972 182 169 2000 10.1002/jez.1401820203 5079084

[B64] Arezo MJ Pereiro L Berois N Early development in the annual fish Cynolebias viarius J Fish Biol 2005 66 1357 1370 10.1111/j.0022-1112.2005.00688.x

